# Oxytocin and Vasopressin Receptor Gene Polymorphisms: Role in Social and Psychiatric Traits

**DOI:** 10.3389/fnins.2015.00510

**Published:** 2016-01-28

**Authors:** Mauricio Aspé-Sánchez, Macarena Moreno, Maria Ignacia Rivera, Alejandra Rossi, John Ewer

**Affiliations:** ^1^Centro Interdisciplinario de Neurociencia de Valparaíso, Universidad de ValparaísoValparaíso, Chile; ^2^Centro de Investigación en Complejidad Social, Facultad de Gobierno, Universidad del DesarrolloSantiago, Chile; ^3^Scuola Internazionale Superiore di Studi AvanzatiTrieste, Italy; ^4^Programa de Doctorado Interdisciplinario de Neurociencias, Facultad de Medicina, Pontificia Universidad Católica de ChileSantiago, Chile; ^5^Department of Psychiatry, Harvard Medical SchoolBoston, MA, USA

**Keywords:** SNP, SSR, psychiatric disorders, dopamine, serotonin, polygenic trait, quantitative traits loci, GWAS

## Abstract

Oxytocin (OXT) and arginine-vasopressin (AVP) are two phylogenetically conserved neuropeptides that have been implicated in a wide range of social behaviors. Although a large body of research, ranging from rodents to humans, has reported on the effects of OXT and AVP administration on affiliative and trust behaviors, and has highlighted the genetic contributions of OXT and AVP receptor polymorphisms to both social behaviors and to diseases related to social deficits, the consequences of peptide administration on psychiatric symptoms, and the impact of receptor polymorphisms on receptor function, are still unclear. Despite the exciting advances that these reports have brought to social neuroscience, they remain preliminary and suffer from the problems that are inherent to monogenetic linkage and association studies. As an alternative, some studies are using polygenic approaches, and consider the contributions of other genes and pathways, including those involving DA, 5-HT, and reelin, in addition to OXT and AVP; a handful of report are also using genome-wide association studies. This review summarizes findings on the associations between OXT and AVP receptor polymorphism, social behavior, and psychiatric diseases. In addition, we discuss reports on the interactions of OXT and AVP receptor genes and genes involved in other pathways (such as those of dopamine, serotonin, and reelin), as well as research that has shed some light on the impact of gene polymorphisms on the volume, connectivity, and activation of specific neural structures, differential receptor expression, and plasma levels of the OXT and AVP peptides. We hope that this effort will be helpful for understanding the studies performed so far, and for encouraging the inclusion of other candidate genes not explored to date.

## Introduction

**Figure 1:**
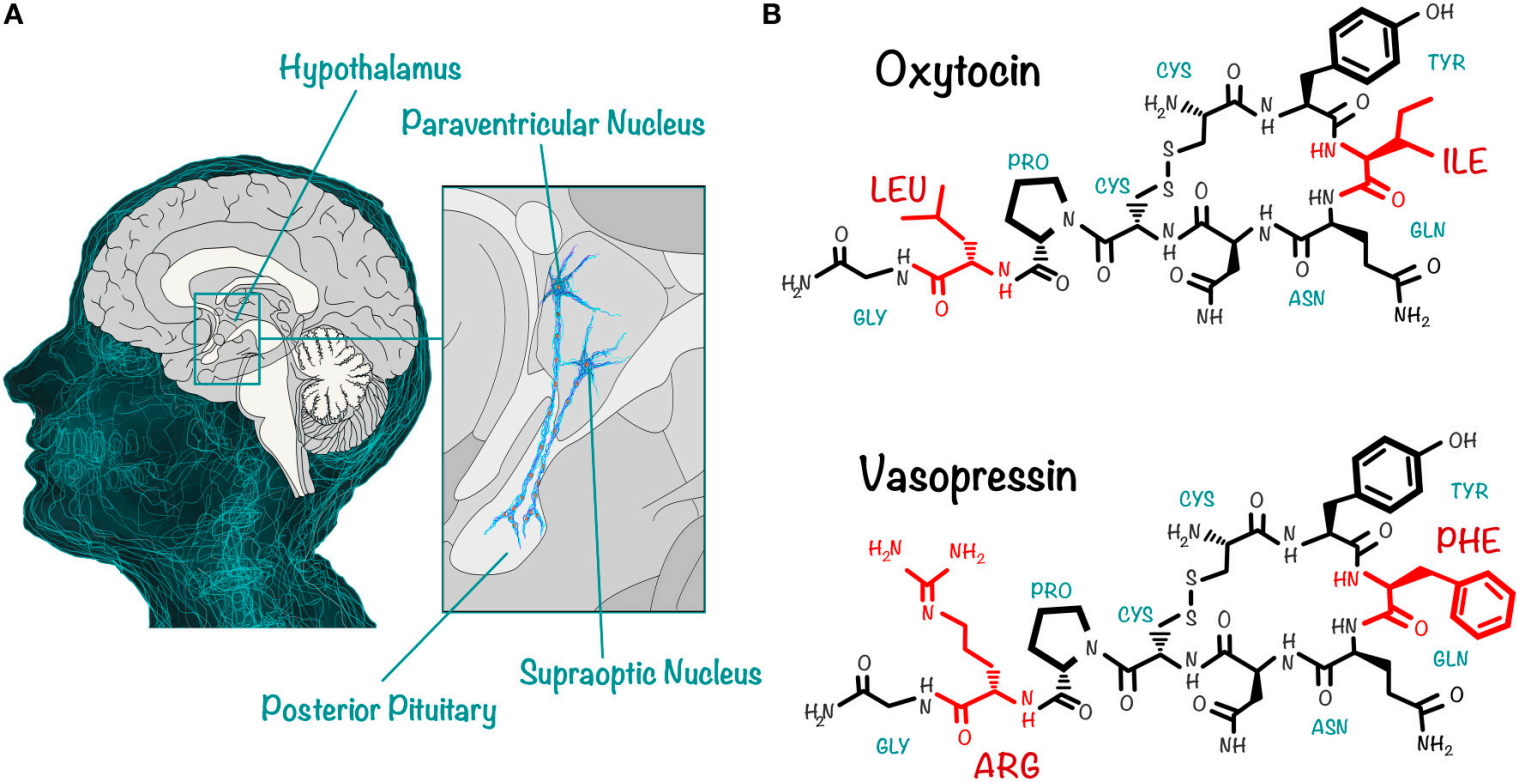
**OXT and AVP are two closely related nonapeptides that exert their action on central and peripheral targets**. **(A)** OXT and AVP are synthesized in the PVN and the SON of the hypothalamus. The peptidergic neurons in these nuclei project axons to the posterior pituitary, from where the peptides are released into the circulation. They act as hormones on peripheral targets, having well-documented actions (uterine contraction and vasoconstriction, for instance). In addition, dendrites of neurons in the PVN and the SON release the peptides directly into the brain, where they act as neurotransmitters or neuromodulators, regulating complex social cognition and behaviors. **(B)** OXT and AVP differ in only two aminoacids: this schematic drawing shows that, whereas the aminoacid sequence of OXT (top) includes an isoleucine at the third and a leucine at the eighth position, that of AVP (bottom) includes a phenylalanine and an arginine in the corresponding positions. Both peptides contain a cyclic six aminoacid ring, because of the disulfide bond formed by two cysteine residues.

Today, the neuropeptides OXT and AVP are, perhaps, the most interesting molecules for social neuroscience (Insel, 2010; Meyer-Lindenberg et al., 2011; Zink and Meyer-Lindenberg, 2012). They are closely related, phylogenetically conserved nonapeptides, which originated more than 700 million years ago (Macdonald and Macdonald, 2010) and differ in only two aminoacids (Caldwell et al., 2008; Insel, 2010; Macdonald and Macdonald, 2010): whereas the aminoacid sequence of OXT includes an isoleucine and a leucine at the third and eighth position, respectively, AVP has a phenylalalanine and an arginine in the corresponding positions (Figure 1). Both peptides contain two cysteine residues that form a disulfide bond, creating a cyclic six aminoacid ring (Caldwell et al., 2008).

OXT and AVP are synthesized in the somas of two groups of neurons located in the paraventricular and supraoptic nucleus of the hypothalamus (Figure 1); they are then transported along axons that project to the posterior pituitary, from where they are ultimately released into the circulation, acting as hormones on peripheral targets (Loup et al., 1991; Meyer-Lindenberg et al., 2011; Zink and Meyer-Lindenberg, 2012). In addition to their well-documented peripheral actions (e.g., that of OXT in uterine contraction and milk ejection, and of AVP in antidiuresis and vasoconstriction; Loup et al., 1991) they also function within the brain as neuromodulators or neurotransmitters. There they act mainly via dendritic release (Landgraf and Neumann, 2004; Ludwig and Leng, 2006; Leng and Ludwig, 2008; Neumann and Landgraf, 2012; Stoop, 2012) to regulate complex social cognition and behaviors, including attachment (Young et al., 1999; Insel, 2010) and social exploration and recognition (Meyer-Lindenberg et al., 2011). Furthermore, there is a growing body of evidence showing that these neuropeptide signaling pathways are impaired in mental disorders associated with social deficits (Meyer-Lindenberg et al., 2011).

This review summarizes findings on the impact of different OXT and AVP receptor polymorphism on social cognition and behaviors, and on some of the most common mental disorders associated with deficits in social function. Our goal is to put special emphasis on the studies that have shed light on associations involving different receptor polymorphism, even if the functional significance of these polymorphisms is currently unknown. In addition, we highlight the importance of polygenic approaches for the fruitful understanding of the OXT and AVP signaling mechanisms and the studies on the effect of intranasal OXT and AVP infusions on psychiatric disorders.

## OXT and AVP receptors and their polymorphisms

**Figure 2:**
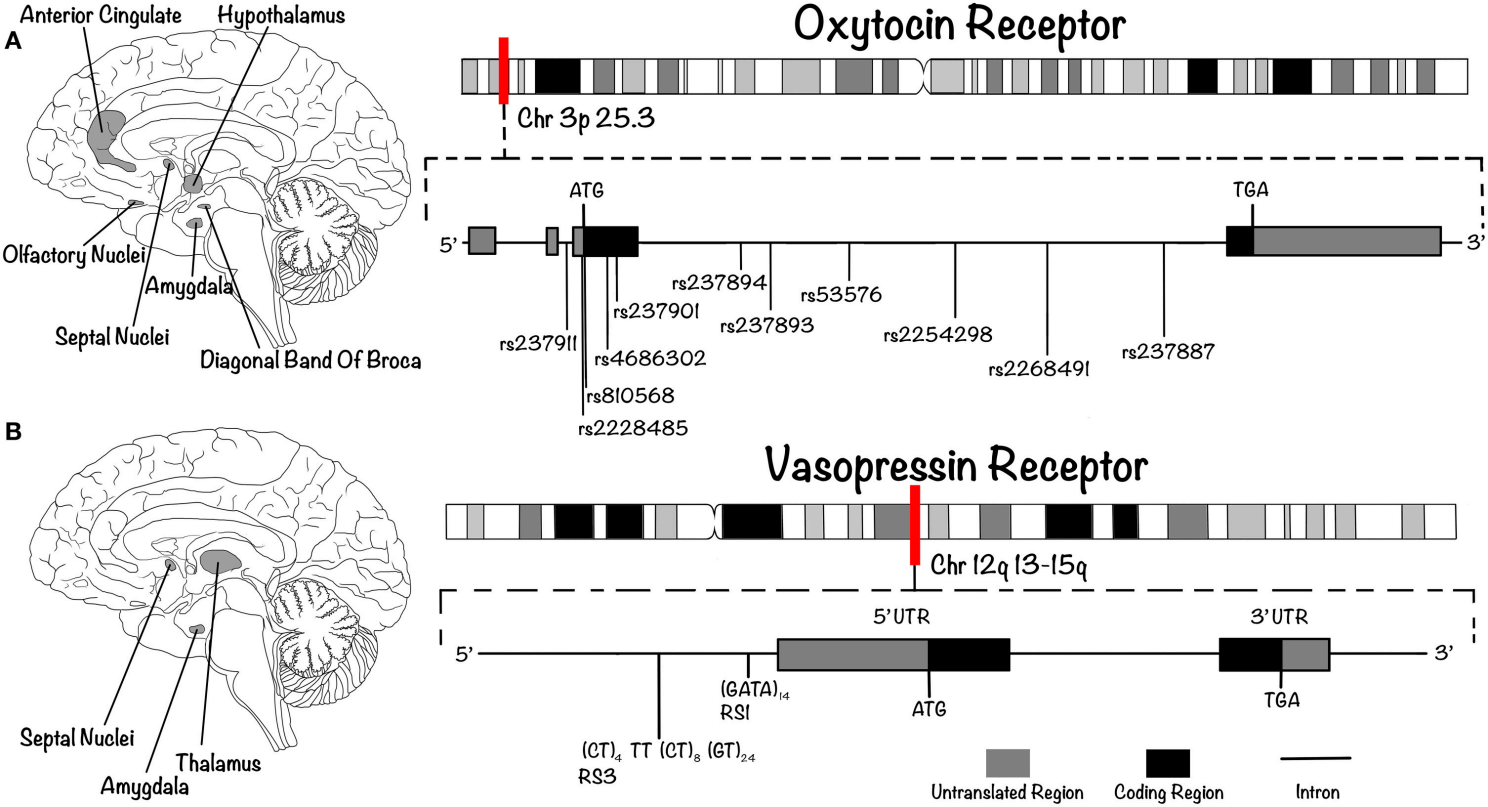
**OXTR and AVPR are G-protein coupled receptor expressed in key structures of the brain**. Their genes present characteristic polymorphisms associated with differences in human social (and pathological) behaviors. **(A)** In the human brain, OXTR is expressed in the basolateral amygdala, the anterior and ventromedial hypothalamus, the olfactory nucleus, the diagonal band of Broca, the septal nuclei and the anterior cingulate (left). Its gene (right) is located on chromosome 3p25.3 (approximate position indicated by red vertical line). It contains four exons and three introns, which include several known SNPs. **(B)** AVPR1A is expressed in the septal nuclei, the thalamus and the basal amygdaloid nucleus (left); the gene encoding this receptor (right) is located on chromosome 12q14 (approximate position indicated by red vertical line). As in the case for OXTR, it contains an intron before the exon that encodes the seventh transmembrane domain. The schematic includes the SNPs (in the case of OXTR) and SSRs (in the case of AVPR) that are reviewed in this article.

OXT and AVP receptors (OXTR and AVPR, respectively) belong to the seven transmembrane domain G-protein coupled receptor super-family. Whereas only one form of OXTR is known, three subtypes of AVPR exist, called AVPR1a, AVPR1b, and AVPR2 (Ebstein et al., 2012). The AVPR1a and AVPR1b subtypes are both coupled to G_q_, and signal via phospholipase C. The inositol triphosphate (IP_3_) produced upon receptor activation induces the mobilization of calcium (Ca^2+^) from intracellular reservoirs; it also causes the release of diacylglycerol (DAG), which increases intracellular Ca^2+^ and activates protein kinase C (PKC). The rise in intracellular calcium levels activates map kinase (MAPK) and calcium/calmodulin-dependent protein kinase II (CAMKII). In addition, activation of this signaling pathway produces a depolarization that leads to the entry of extracellular Ca^2+^ via voltage-gated calcium channels (Thibonnier, 1992; Thibonnier and Schork, 1995; Thibonnier et al., 2000). AVPR1a and AVPR1b are expressed in several tissues and organs, including platelets, the adrenal cortex, kidney, spleen, smooth muscle, endothelium, and adipocytes (Thibonnier, 1992; Thibonnier and Schork, 1995; Thibonnier et al., 2000). In the human brain, they are expressed in the lateral septum, thalamus, basal amygdaloid nucleus, and brainstem (Figure 2) but, interestingly, not in the cortex (Meyer-Lindenberg et al., 2009). The AVPR2 subtype, on the other hand, is coupled to G_s_, which activates adenylyl cyclase (AC) and causes the production of cAMP and the activation of protein kinase A (PKA). This receptor subtype is expressed on the basolateral membrane of the colleting duct in the medullary portion of the kidney (Thibonnier et al., 2000). The single OXTR, on the other hand, is coupled to G_q_, activating the same intracellular pathway as AVPR1a and AVPR1b. It is expressed in the uterus, the mammary gland, the ovary, lactotroph cells and, in the brain, in the central and basolateral amygdala, medial preoptic area, anterior and ventromedial hypothalamus, olfactory nucleus, vertical limb of the diagonal band of Broca, ventrolateral septum, anterior cingulate, and hypoglossal and solitary nuclei (Figure 2; Loup et al., 1991; Boccia et al., 2013). No expression has been reported in the hippocampus (including CA2 and CA3), parietal cortex, raphe nucleus, nucleus ambiguus or pons (Boccia et al., 2013).

Interestingly, there is some evidence for the formation of heterodimers between AVPR1a, AVPR2, and OXTR (Cottet et al., 2010). Formation of such heteromeric receptors could have important *in vivo* implications, and possibly confound the interpretation of hormonal and neuromodulatory actions (Devost and Zingg, 2003; Cottet et al., 2010). More importantly, despite the highly conserved protein structure of the peptides and of their receptors, it is noteworthy (particularly when extrapolating to humans findings from research done on animals) that their corresponding gene structure and spatial pattern of expression presents species-specific variations, as shown, for instance, in the sequence and brain expression of *AVPR1a* in monogamous vs. non-monogamous vole species (Hammock and Young, 2004, 2005). The variations in gene structure of OXTR and AVPR mostly occur in the form of genetic polymorphisms, which have been proposed to underlie inter- (Young et al., 1999) and intraspecific (Young et al., 1999; Knafo et al., 2008) differences in expression levels (Tansey et al., 2011) and spatial expression patterns (Young et al., 1999; Knafo et al., 2008) in the brain.

### Types of polymorphisms

A genetic polymorphism is the occurrence of two or more genetic variants for a particular locus in the DNA sequence (i.e., alleles) within a population. Polymorphisms can be classified depending on the types of changes observed in the nucleotide sequence. One of the most common is a change at the level of single nucleotides (single nucleotide polymorphism, SNP). SNP's located in coding regions of the DNA can in turn be classified as nonsynonymous and synonymous, depending on whether or not they alter the amino-acid sequence of the resulting protein, respectively. Finally, a non-synonymous SNP can change the protein's amino-acid sequence (i.e., missense mutation) or result in a truncated protein (i.e., nonsense mutation). SNPs located in noncoding regions can affect the levels of expression and the spatial expression of a gene, and can therefore also alter gene action (Plomin et al., 2008). Several SNPs can occur simultaneously at different locations within a given gene and tend to segregate together. Such linked SNPs are called haplotype blocks, or simply haplotypes. The occurrence of these blocks is useful for investigating the genetic bases of common diseases, as it can reduce the number of variants from 500,000 to around 350,000 (Plomin et al., 2008).

Another common type of polymorphism is microsatellites, or tandem simple sequences repeats (SSRs), which are sequences of about 2–5 bp in length that are repeated, in tandem, between 5 and up to 50 times. In this case, alleles differ in the number of SSR repeats that they contain. Despite the fact that most of the microsatellites in the human genome are located in non-coding regions, and are thus usually considered to be evolutionary neutral, they can nevertheless cause phenotypic differences (Li et al., 2004), including for neurological disorders in humans (Amir et al., 1999).

## Inheritance studies on human behavior

Studies on behavioral genetics have consistently shown that heritability (the proportion of phenotypic variance attributable to genotypic variance) plays a role in determining human complex social traits. According to Plomin et al. (2008), behaviors can depend on genotype in (at least) three ways: (i) passively, where children inherit from their parents an environment that is correlated with the children's genetic propensities; (ii) evocatively, where children evoke reactions from other people on the basis of their genetic propensities, and (iii) actively, by selecting, modifying, and constructing experiences correlated with their genetic propensities. Thus, a child inheriting a “depressive” allele could also inherit a depressor environment from her depressive parents (passive); act apathetically, making others avoid her (evocative); and develop cognitive biases, viewing the life through gray glasses and creating, in this way, her own depressing reality (active). These genetic influences on environmental measures have been called genotype-environment correlations (Plomin et al., 2008). In addition, there exist genotype-environment interactions, where the effect of environment on some traits depends on the genotype and, conversely, the effect of a given genotype depends on the environment. Phenotypes are, in this way, more than the sum of independent genetic and environmental factors: for instance, variations in socially important behaviors are sensitive to variations in genotypes (Plomin et al., 2008). The early approaches that shed light on the genetic influences on human social behavior, namely the linkage/association studies and the quantitative traits loci (QTL) approach, are described below.

### Linkage and association studies

In classical linkage studies, heritability is measured on the basis of correlations found among relatives. These studies typically analyze large pedigrees of families presenting a characteristic trait, and, ideally, include monozygotic (MZ) and dizygotic (DZ) twins. Because MZ twins have exactly the same genotype, whereas DZ twins on average share only about 50% of their genetic material, if genetic differences account for phenotypic differences, then MZ twins should exhibit higher correlation than DZ twins in any particular trait (Cesarini et al., 2008; Plomin et al., 2008). Furthermore, if MZ and DZ twins live in a shared environment, then these correlations are useful for estimating the relative influence of genetic vs. environmental factors on phenotypic variation (Cesarini et al., 2008; Plomin et al., 2008). A classical success story of this approach is in the analysis of a five-generation pedigree of a family with members afflicted by Huntington disease, which lead to the identification of the CAG repeat associated with most cases of this disease (Walker, 2007; Plomin et al., 2008). Alternatively, sib-pair linkage analyses can be carried out, in which case a small number of relatives is considered, but in a large number of families. In both approaches, the researcher is interested in looking for a marker that is co-inherited with the trait of interest: however, these approaches cannot detect associations in which genes have small effects, as is the case for most complex behaviors or disorders (Risch, 2000; Plomin et al., 2008). In addition, single-gene disorders tend to be rare, whereas many important multifactorial traits or diseases, including autism, depression, schizophrenia and others affecting human social behavior, occur at much higher frequencies in the population, and also show high heritabilities (Risch, 2000; Plomin et al., 2008).

Association studies, rather than relying on the incidence of a disease (and its associated markers) within families, test whether the frequencies of specific alleles or genotypes differ between groups such as affected individuals vs. controls, or among individuals with extreme scores on a quantitative trait (Plomin et al., 2008). These studies have classically been restricted to a few “candidate” genes (see for instance, Knafo et al., 2008; Rodrigues et al., 2009; Thompson et al., 2011; Luo et al., 2015a,b). The influence of a given allele is then quantified, for instance, using the odds ratio, which is calculated as the ratio of the frequency of an allele in affected individuals divided by the frequency of this same allele in the control sample. By this measure, an odds ratio of 1.0 means that there is no difference in allele frequencies between affected individuals and controls, whereas odds ratios larger than 3.0 is considered to represent a large effect (Plomin et al., 2008; Brookfield, 2010).

### Pitfalls of linkage and association studies

In any association study, it is critical that the groups be well-matched in terms of ethnicity, gender, and age (Plomin et al., 2008; Brookfield, 2010). If not, the study could identify spurious factors, which are simply due to differences between the groups (Plomin et al., 2008; Brookfield, 2010). For instance, if a disease is more prevalent in one of two ethnically different populations, then any genetic variant that is more common in one of these populations will show an association with a disease simply because of differences in ethnicity. This could be the case for some reports reviewed here, which show inconsistent results but were based on different ethnics groups.

### Quantitative traits loci (QTL)

In a classical monogenic approach, the presence or absence of a trait follows a dichotomous distribution. In the case of qualitative diseases (diseases where the trait is either present or not), a single allele would be necessary and sufficient for the trait to be expressed (Plomin et al., 2008; Brookfield, 2010). Currently, around one thousand single-gene qualitative disorders are known (Plomin et al., 2008). However, when the penetrance is very low, it is virtually impossible to map genes using pedigrees (Plomin et al., 2008; Brookfield, 2010); more importantly, most human social and pathological behaviors are better described as a quantitative and continuous, rather than as a discrete, qualitative variable (Plomin et al., 2008). A polygene (or quantitative gene), on the other hand, is a group of usually pleiotropic genes, which together influence a phenotypic trait, leading to additive, non-epistatic effects (Plomin et al., 2008; Brookfield, 2010). Thus, a quantitative trait is a phenotype that varies in degree and can be attributed to polygenic effects, i.e., the product of two or more genes. Each gene is inherited in a Mendelian fashion, but the additive effect of several genes results in phenotypes that approach a normal distribution within the population (Plomin et al., 2008; Brookfield, 2010). If we include into this model the effects of the environment, then we are dealing with multifactorial traits, where the presence or absence of a trait or disease is influenced by many genetic differences as well as by the environment (Plomin et al., 2008). This approach predicts a continuum of genetic risk, in which diagnosed subjects could be extreme cases that differ quantitatively, but not qualitatively, from normal cases (Plomin et al., 2008).

## Role of OXTR and AVPR in social behaviors

### Studies in rodents

Reports implicating OXT and AVP in the control of mammalian social behavior first appeared in the early 90's. For instance, Insel and Shapiro (1992) showed that the distribution of OXTR in the brain of voles differed between monogamous (prairie) and polygamous (montane) species. Likewise, AVPR distribution also differed between these species, most likely because of differences in promoter structure (SSRs polymorphisms; Young et al., 1999). Most interestingly, intracerebroventricular injections of AVP increased affiliative behaviors in the monogamous prairie vole, but not in the promiscuous montane species (Young et al., 1999), and transgenic mice expressing the prairie vole variant of AVPR also showed increased affiliative behaviors in response to AVP injections (Young et al., 1999). Collectively, these findings suggest that the different pattern of AVPR expression is responsible for the different affiliative behaviors of these two closely related species of voles, as well as for their behavioral responses to AVP.

Since these early studies, a large amount of research has been carried out on these two so-called “prosocial neuropeptides,” most of them using the powerful techniques available in rodent models (Nakajima et al., 2014; Sala et al., 2015; Yan et al., 2015). Regarding OXT, for instance, Nakajima et al. (2014) demonstrated that blocking neurotransmission from medial prefrontal cortex (mPFC) interneurons that express OXTR (specifically, somatostatin neurons) caused female mice to lose interest in male mice during the sexually receptive phase of the estrous cycle (Nakajima et al., 2014). Similarly, behavioral despair (a behavior associated with depression) was shown to enhance OXT synthesis and secretion in the paraventricular nucleus, supra-optic nucleus, frontal cortex, amygdala, and hippocampus, as well as OXT release from the posterior pituitary into the blood (Yan et al., 2015). Finally, and as a way of example, *OXTR* null mice (*OXTR* ^−∕−^) display increased aggression and autistic-like deficits in social behaviors, such as a resistance to changes in a learned pattern of behavior (comparable to the restricted interest and repetitive behaviors associated with autism; Sala et al., 2015). Furthermore, this study suggests that intracerebral administration of both OXT and AVP reverts the social and learning defects by acting on AVPR1a receptors (Sala et al., 2015). Insights have also been obtained into the possible functionality of some of the *AVPR* alleles, including the impact of SSRs on the pattern of AVPR expression in the brain (Young et al., 1999; Hammock et al., 2005), transcriptional regulation (Hammock and Young, 2004, 2005), and promotor activity (Tansey et al., 2011), all of which are associated with differences in social behaviors, including affiliation (Insel, 2010), aggression (Beery et al., 2008), mating (Young et al., 1999; Caldwell et al., 2008), and anxiety-like behaviors (Hammock et al., 2005; Neumann and Landgraf, 2012).

Regarding psychiatric models, OXT administration has been shown to mimic the effects of antipsychotic drugs. For example, subcutaneous OXT administration in rats reduces prepulse inhibition, a behavioral measure of sensorimotor gating in which a startle response to a stimulus is reduced when it is preceded by a weaker stimulus (Feifel and Reza, 1999; Feifel et al., 2012). These results are relevant since prepulse inhibition is a neurological phenomenon commonly seen in schizophrenic patients (Powell et al., 2009; Ratajczak et al., 2013) thereby suggesting that OXT could produce antipsychotic-like central effects.

Notwithstanding the fact that results from rodents are not easy to extrapolate to primates (for instance, Fink et al. (2006) found that polymorphisms upstream of *AVPR1a*, which have been associated with differences in social bonding, are evolutionarily distinct between primates and rodents), a substantial body of evidence obtained (initially) through linkage and association studies suggests that *OXTR* and *AVPR* polymorphisms could be part of the genetic sources of the heterogeneity observed in social traits and psychiatric disorders (Meyer-Lindenberg et al., 2011). The next sections describe the structure of the OXTR and AVPR genes and summarize some of the monogenic studies supporting the role of polymorphisms in these genes in human social and pathological behavior.

### Studies in human

In humans, the 17Kb *OXTR* is located on chromosome 3p25.3 and contains four exons and three introns, one of them containing more than twelve known SNPs (Inoue et al., 1994). In the case of AVP, the genes encoding the 3 receptors (*AVPR1a, AVPR1b*, and *AVPR2*) are located on chromosomes 12q14, 1q32, and Xq28, respectively. As is the case for *OXTR*, all contain an intron before the exon that encodes the seventh transmembrane domain (Seibold et al., 1992; Sugimoto et al., 1994; Thibonnier et al., 1996). The majority of polymorphisms studied in OXTR gene are SNPs (Figure 2), and some of them appear to be strongly associated with variability in social traits. One of the most studied is rs53576, which consists of a G to A change within the third intron of *OXTR.* In the case of *AVPR1*, the most studied polymorphisms are SSRs, such as RS3 and RS1 (Figure 2), the former located upstream of the transcription start site and the latter located in a coding region 553 bp downstream from the start site (Meyer-Lindenberg et al., 2011).

#### Monogenic studies

In this section, we summarize the results from monogenetic studies that have investigated the relationship between polymorphisms in OXTR and AVPR and human social and pathological behaviors. These analyses are summarized in Table 1.

**Table 1:** **OXTR and AVPR polymorphisms associated with social and pathological behaviors**.

**Trait**	**Gene**	**Polymorphism studied**	**Species/Ethnicity**	**Associated Genotype or Haplotype**	**Behavioral phenotype**	**Brain/Biological phenotype**	**Interactions with other gene or variable**	**References**
Aggression	AVPR1b	rs35369693	147 Europeans, 10 African Canadians, 2 East Asians, and 18 of mixed ethnicity	C allele	Increased aggression	–	Haplotypes with rs28676508	Zai et al., 2012; Luppino et al., 2014
Autism	AVPR1a	RS1	Irish	Short allele	Autism	Increased activity in the left amygdala in a non-clinical sample	Possibly reduced transcription of AVPR1A	Tansey et al., 2011
Autism	AVPR1a	RS1; RS3	94 Caucasian, 7 African-American, 8 Asian-American and 6 Hispanic (17 females)	RS3	In linkage disequilibrium in autism (not significant after bonferroni correction)	–	–	Kim et al., 2002
Autism	AVPR1a	RS1; RS3 and AVR	Not specified (*n* = 128 probands, 3 females)	AVR	Transmission desequilibrium in autism; Moderate linkage desequilibirum and association with some scores related to autism	–	Haplotypes involving alleles of the three polymorphisms	Yirmiya et al., 2006
Autism	OXTR	rs2254298 and rs53576	57 Caucasian trios	rs2254298 G	Overtransmited in autism	Phenotypic heterogeneity	Haplotypes with an as yet unidentified susceptibility variant	Jacob et al., 2007
Autism	OXTR	rs2254298, rs53576, rs237893, rs237894, rs237911, rs237901, rs810568, rs2228485	195 Chinese Han family trios (21 females)	rs2254298 A and rs53576 A; haplotypes involving rs53576	Displayed a preferential transmission in Autism	–	–	Wu et al., 2005
Autism	AVPR1b	rs2254298, rs53576, rs237893, rs237894, rs237911, rs237901, rs810568, rs2228486	89 Swedish, 89 Belgians in the patients sample (122 females); 88 Swedish and 89 Belgiansin the control sample (117 females)	G allele for s3 in Swedish sample; G allele for s5 in the Belgian sample; A-T-C-A-G Haplotype in both samples	Recurrent major depression; Overrepresented in controls; Overrepresented in controls	–	Ethnicity	Van West et al., 2004
Depression	OXTR	rs2254298	92 Caucasian adolescent girls	Heterozygous	Highest levels of symptoms of depression, physical and social anxiety, if they presented history of maternal depression	–	Maternal history of recurrent major depressive disorder	Thompson et al., 2011
Depression	OXTR	rs2254298	Not specified (first cohort composed of 1983 pregnant females)	G Homozygous	Overrepresented in depressed mothers and families (including fathers)	Lower salivary OXT	–	Apter-Levy et al., 2013
Depression	OXTR	rs53576 (G/A)	413 Caucasian, 18 Asian, 5 Maori/Pacific Islander, 2 Aboriginal and 1 other (261 females)	A Carriers	Higher depressive symptoms if mother has been depressed	–	Association between maternal depression in early childhood and youth depressive symptoms in adolescence	Thompson et al., 2014
Depression	OXTR	rs53576 (G/A)	167 White, 34 Black, 23 Asian, 17 Arab, 16 South Asian, 6 South East Asian, 4 Latin American, 17 other (213 females)	G Carriers	Increased depressive symptomatology if suffered childhood maltreatment	–	Childhood maltreatment	McQuaid et al., 2013
Depression	OXTR	rs53576 (G/A)	128 White female	G Carriers	More emotionally sensitive (lower self-esteem) in response to social ostracism	Altered blood pressure and cortisol levels following rejection for G homozygous	–	McQuaid et al., 2015
Depression	OXTR	rs53576; rs2254298	99% Caucasians (71% females; *n* = 268)	No association found	Antidepressant treatment resistance, response or remission	–	Cyclooxygenase-2 (COX-2), was associated with antidepressant treatment resistance	Mendlewicz et al., 2012
Depression	OXTR	rs53576	43 clinically depressed and 42 healthy female subjects	Exon 1 and 2 methylation patterns	Decreased methylation state were associated with depression	Regulate DNA methylation throughout the whole genome, depending on early environment	Specific genotypes can regulate DNA methylation	Chen et al., 2015
Empathy	OXTR	rs2268491; rs2254298	Non-clinical Chinese subjects (46 males, 55 females)	CT higher than CC; CT higher than TT	Cognitive empathy	–	Gender- dependent	Wu et al., 2012
Empathy	OXTR	rs237887; rs4686302	Non-clinical Chinese subjects (46 males and 55 females).	A Carriers; T carriers	Higher Emotional empathy	–	Gender-dependent	Wu et al., 2012
Empathy	OXTR	rs53576 (G/A)	35% Caucasian, 41% Asian, and 24% others. (n =192)	G Homozygous	Lower levels of dispositional stress reactivity, exhibit greater empathy	Lower heart rate reactivity to a startle anticipation task	–	Rodrigues et al., 2009
Empathy	OXTR	rs53576 (G/A)	No specified (*n* = 1532)	G Homozygous; A Homozygous	Greater in-group bias in implicit attitudes; Less motivation to reduce out-group member pain	Higher ACC and SMA activity; Higher NAc activity	–	Luo et al., 2015a
Empathy	OXTR	rs53576 (G/A)	Chinese students (*n* = 1536, 710 females)	G Homozygous	Showed a stronger association between empathy and interdependence	Insula, amygdala and superior temporal gyrus	Associated with racial in-group	Luo et al., 2015b
Harm avoidance	OXTR	rs53576 (G/A)	Chinese Han (*n* = 290, 154 females)	A Homozygous	Increased harm avoidance	Smaller amygdala volumes bilaterally Reduced resting-state functional coupling between the prefrontal cortex and amygdala bilaterally in females	Gender- dependent	Wang et al., 2014
Neuroticism	OXTR	rs53576 (G/A)	Chinese Han (*n* = 82, 45 females)	G Carriers; AA	MPI neuroticism scores correlated with DAT and OXT interaction (AA)	Lower striatal DAT availability; Negative correlation between DAT availability and OXT levels in G carriers	–	Chang et al., 2014
Prosocial Behavior	AVPR1a	RS1 and RS3	Non specified (*n* = 203, 101 females) (*n* = 15 for post mortem studies)	Long (327–343 bp) alleles	Larger allocations in a dictator game and higher scores in self-reported altruism scales	Increased mRNA hippocampal levels	–	Knafo et al., 2008
Prosocial Behavior	OXTR	rs53576 (G/A)	108 European - Americans males	G Homozygous	Higher trust behavior	–	–	Krueger et al., 2012
Schizophrenia	AVPR1a	RS3	Russians (291 patients, 49% females; 302 control group, 59% females)	327 bp allele	Schizophrenia's negative symptoms; facial affect recognition	–	–	Golimbet et al., 2015
Schizophrenia (Attention)	AVPR1a	RS1; RS3	Non specified (*n* = 113, 75 female)	Long alleles (>325 bp)	Promoter repeat region in partially molding social behavior in both animals and humans	Greater levels of prepulse inhibition	Gender (stronger association in male)	Levin et al., 2015
Stress	OXTR	rs53576 (G/A)	White (74.9%), Black (8.92%), Hispanic (8.92%), and other (52.3% female)	A carriers; G Homozygous	Increased Post traumatic symptoms in negative social environment, independently of economic stress; Elevated post traumatic symptoms in negative social environment if economic stress exists	–	Economic stress	Thompson et al., 2011
Stress	OXTR	rs53576 (G/A)	173 European participants, 15 mixed, and 6 “other.” All males	G carriers	Attenuated anticipatory stress response if they received social support	Lower cortisol responses under stress after social support	Social support	Chen et al., 2011
Stress	OXTR; AVPR1a	rs53576; RS1	37% Asian-American, 2% African-American, 23% European-American, 16% Latin American, 7% mixed ethnicity and 15% other. (*n* = 172, 60% females)	G Homozygous; 320 bp	Women with higher poststressor OXT levels reported more positive affect feelings; Men with high levels of poststressor AVP reported more anger and hostility feelings	–	Gender and poststressor levels of OXT and AVP	Moons et al., 2014

##### Prosocial behavior

An interesting line of research has shown that humans display several “prosocial” behaviors, including trust, generosity and altruism. These result are of great interest to fields such as Neuroeconomics (Fehr and Camerer, 2007; Ebstein et al., 2009), because the assumption of neoclassical economics is that individuals always act to maximize a utility function which depends exclusively on their own material gain (Fehr and Schmidt, 1999). Because of a growing body of evidence that does not support this hypothesis, this utility function has had to be modified to include other-regarding preferences (Rabin, 1993; Fehr and Schmidt, 1999; Dufwenberg and Kirchsteiger, 2004).

Two independent studies of monozygotic and dizygotic twins, one in Sweden and one in the United States, have shown that there is a significant heritability of prosocial behaviors. Both studies were reported by Cesarini et al. (2008), who estimated that correlations in trust behavior, as measured in a classical Trust game, are 0.13 and 0.25 in the case of North Americans and Swedish MZ twins, respectively, vs. 0.07 and 0.01 in both populations of DZ twins. The same applies for trustworthiness: MZ were correlated in 0.26 (North Americans) and 0.29 (Swedish), vs. 0.06 (North Americans) and 0.18 (Swedish) for DZ. The heritability estimate is 10% (with a 4–21% confidence interval) for the U.S. study and 20% (with a 3–38% confidence interval) for the Swedish study (Cesarini et al., 2008). Interestingly, unshared environmental variation has a greater impact on phenotypic variation than genetic variation. In another study, the group of Cesarini found that strategies and fundamental economic preference parameters are moderately heritable, with estimates of heritability ranging from 18 to 42% (Cesarini et al., 2009) and, importantly, has suggested that these traits have a polygenic architecture, with the heritable variation being explained by many genes with small effects (Benjamin et al., 2012).

Several association studies are also beginning to shed light on the molecular basis of variations in these so-called “prosocial” behaviors (Kosfeld et al., 2005; Zak et al., 2007; Israel et al., 2009; Ebstein et al., 2010; Krueger et al., 2012; Radke and De Bruijn, 2012). In one of the first reports, Knafo et al. (2008) classified the *AVPR1a* RS3 SSRs of participants into short (308–325 bp) and long (327–343 bp) versions, and found, using the Dictator game, that subjects with the short version allocated significantly fewer tokens to others than did participants with the long versions. This was confirmed and validated using the family-based association test and two self-report scales (the Bardi-Schwartz Universalism and Benevolence Value-expressive Behavior scales). Interestingly, long *AVPR1a* RS3 repeats were associated with higher levels of hippocampal *AVPR1a* mRNA levels in human *post-mortem* studies, as compared to those measured in individuals bearing the short RS3 repeats (Knafo et al., 2008). Along a similar line, Krueger et al. (2012) reported that individuals homozygous for the OXTR rs53576 G allele shows higher trust behavior (not just a general increase in trustworthiness or risky behaviors) than do A carriers (i.e., individuals presenting at least one A allele in this SNP; Krueger et al., 2012). Similarly, OXT infusions increase trust (Kosfeld et al., 2005), generosity (Zak et al., 2007), and decrease adherence to social norms (Radke and De Bruijn, 2012).

In addition, *AVPR1b* alleles have been associated with aggression levels in children. Luppino et al. (2014) reported a significant relationship between the presence of the minor C allele in SNP rs35369693 of *AVPR1b* and teacher-rated reactive aggression (Luppino et al., 2014). Similarly, Zai et al. (2012) found that this same SNP, as well as haplotypes containing rs35369693 and rs28676508 (both in *AVPR1b*), were also associated with higher child aggression (subjects that scored at or above the 90th percentile on the aggression subscales of both the Child Behavior Checklist and the Teacher's Report Form; Zai et al., 2012).

##### Empathy

Empathy is a social behavior defined as a capacity to share and understand the feelings of others (De Vignemont and Singer, 2006). Rodrigues et al. (2009) reported that subjects homozygous for the G allele of *OXTR* SNP rs53576 exhibit greater empathy compared to A carriers, as measured by the “Reading the Mind in the Eyes” task. Luo et al. (2015a) compared the behavior and the fMRI responses of individuals when they observed painful or non-painful stimulations of other subjects, who were categorized by racial ingroup/outgroup criteria. They found that individuals that were homozygous for the G variant showed increased activity in the anterior cingulate cortex (ACC) and supplementary motor area (SMA) in response to racial ingroup members' pain, as compared to individuals homozygous for the A variant. By contrast, individuals homozygous for the A allele showed increased activity in structures of the reward system, specifically in the nucleus accumbens, when they watched a racial outgroup member in pain. This opposite response also predicts the participants' attitudes and behavior: the racial ingroup bias in ACC/SMA activity was positively correlated with participants' racial ingroup bias in implicit attitudes (specifically, they made more associations between Asian vs. Caucasian faces and concepts of “good” vs. “bad,” as measured by the Implicit Association Test), whereas nucleus accumbens activity showed a negative correlation with participants' motivations to reduce racial outgroup members' pain.

Noticing the different results obtained between Eastern vs. Western subjects in studies on empathy, this same group also investigated whether the *OXTR* rs53576 SNP interacts with the interdependence trait (i.e., how people view themselves in relation to others) to modulate human empathy. They found a stronger association between interdependence and empathy in G carriers, as compared to A homozygotes (Luo et al., 2015b). In addition, they used fMRI to measure the neural responses elicited by the suffering of others, finding stronger associations between interdependence and empathic neural responses in G/G, as compared to A/A genotypes, in brain structures such as the insula, amygdala, and the superior temporal gyrus Luo et al. (2015b).

Other studies have investigated the association between other common *OXTR* SNPs and empathy. Emotional empathy is defined as the ability to respond with an appropriate emotion to another's mental states (De Waal, 2008; Shamay-Tsoory et al., 2009). Wu et al. (2012) found an association between emotional empathy and *OTXR* variation at the rs237887 SNP (with A allele subjects scoring higher than those with the G allele), and the rs4686302 SNP (with T allele subjects scoring higher than those with the C allele). By contrast, cognitive empathy, the capacity to understand another's perspective or mental state (De Waal, 2008), showed associations with SNP polymorphisms rs2268491 (with carriers of the C/T variant scoring higher than those of the C/C) and rs2254298 (with C/T carriers scoring higher than T/T carriers; Wu et al., 2012).

##### Autism

Autism is a neurodevelopmental disorder characterized by abnormalities in social relationships, communication deficits, and restricted interests (Plomin et al., 2008), with a prevalence of 3–6 cases per 10,000 people. Its prevalence is four times higher in males than in females (Freitag, 2007; Plomin et al., 2008). Although the risk of autism in offspring of autistic parent(s) is small (about 5%; Freitag, 2007; Plomin et al., 2008), it is, nonetheless, 100 times greater than the rate of autism in the general population (Freitag, 2007; Plomin et al., 2008).

A couple of studies have investigated the association between SNPs rs53576, rs2268498, and autism, with rather confusing results. In one of two family-based association tests (FBAT; Horvath et al., 2001), Wu et al. (2005) found, in a study involving Chinese Han individuals, a significant association between autism and individual variants of *OXTR* SNPs rs53576 and rs2254298, as well as with haplotypes involving rs53576 (specifically, A-A-T-A in rs53576, rs2254298, rs2228485, and rs237911, respectively). By contrast, Jacob et al. (2007), using Caucasian individuals, found a significant association between autism and polymorphism at the rs2254298 SNP, but not rs53576. Moreover, they found that the overtransmission (i.e., a higher likelihood that the risk allele is transmitted to the affected child, as compared to the non-risk allele) of the G allele was more strongly associated with the autistic disorder group, again in contrast to the study involving Han patients (Wu et al., 2005), which, as noted above, reported the overtransmission of the A allele (Jacob et al., 2007). In addition, there are some reports associating the short alleles of SSR RS1 in *AVPR* with autism: Tansey et al. (2011), for instance, found that the short alleles of RS1 show a weak association with autism in an Irish population, consistent with a previous study by Kim et al. (2002).

##### Schizophrenia

Schizophrenia is perhaps the most studied disorder of behavioral genetic research in psychopathology (Plomin et al., 2008). Its typical symptoms include delusions, hallucinations (especially auditive), disorganization of speech and behavior, and negative symptoms (i.e., loss of normal processes) like flat affect and avolition (Gottesman, 1991; Plomin et al., 2008). It is estimated that about 1% of the population is afflicted by the disorder, which presents high heritability (about 48% in identical twins; Gottesman, 1991; Plomin et al., 2008). Interestingly, although it runs in families, the particular subtype (catatonic, paranoid, disorganized) does not (Plomin et al., 2008).

There exists a small but significant association between schizophrenia and polymorphisms in the Neuregulin I gene (Lewis et al., 2003; Plomin et al., 2008), which is involved in the development of the nervous system (Harrison and Law, 2006; Plomin et al., 2008). Although there is currently no evidence of an association between polymorphisms in *OXTR* and *AVPR*, and schizophrenia, there are reports of associations between *OXTR* and *AVPR* polymorphisms and traits associated with the disease, such as attention (the main cognitive domain that is impaired in this disorder; Powell et al., 2009; Ratajczak et al., 2013) and negative symptomatology. Golimbet et al. (2015) showed that the *AVPR1A* 327bp SSR in RS3 is associated with negative symptoms (as measured using the Positive and Negative Symptoms Scale, PNSS), and tended to be linked with patient facial affect recognition, probably impacting social phenotypes of schizophrenia. In addition, Levin et al. (2015) used a robust family-based strategy to show that longer RS3 alleles were associated with greater levels of prepulse inhibition, a classical paradigm designed to measure attention. This association was stronger in males.

##### Depression

Depression is a state of low mood and aversion to activity, with characteristic symptoms including anhedonia, overeating or loss of appetite, insomnia, excessive sleep, fatigue, aches, pains, digestive problems, and reduced energy (McGuffin et al., 2003; Plomin et al., 2008). Subjects suffering severe depression may contemplate, attempt to, or commit suicide (McGuffin and Katz, 1989; Plomin et al., 2008), emphasizing its societal importance. The familial risk of major depression and of bipolar disorder is about 9%, as compared to 3 and 1% in control samples for depression and bipolar disorder, respectively (McGuffin et al., 2003; Plomin et al., 2008).

Some reports have shown heritability of depression-protective alleles and depression-related traits. Consistent with an interpersonal perspective on depression (Joiner and Coyne, 1999), Apter-Levy et al. (2013) found that, in the families of depressed mothers, salivary OXT levels was lower in mothers, children and also in fathers, as compared to control families. Children, in addition, had lower empathy and social engagement levels, and 61% of the children displayed axis I disorders, mainly anxiety and oppositional defiant disorder, compared to 15% of children of nondepressed mothers. The *OXTR* rs2254298 SNP homozygous for the G variant was overrepresented in depressed mothers and their families (including fathers), and correlated with lower salivary OXT. The presence of a single A allele in this SNP in depressed mothers markedly decreased the risk psychopathology in the child (Apter-Levy et al., 2013).

Another study found that youth possessing at least one A allele of the *OXTR* rs53576 SNP, whose mothers had had a history of depression (as measured at age 15, using the SCID-I scale), exhibited the highest levels of depressive symptoms at age 15 (as measured by the Beck Depression Inventory II; BDI-II), showing that SNP rs53576 acts as a moderator variable in the transmission of maternal depression from mothers to their children (Thompson et al., 2014). In addition, under high levels of childhood maltreatment (as reported by the Childhood Maltreatment Questionnaire) only carriers of the G allele of SNP rs53576 presented increased depressive symptomatology (as measured by BDI) when compared to those with the A/A genotype (McQuaid et al., 2013). Moreover, carriers of the G allele were more emotionally sensitive (lower self-esteem) in response to social ostracism (measured by the Social Ostracism and Mood Scale), and showed altered blood pressure and cortisol levels following social rejection induced by the Cyberball task, a well-established computerized game used to induce feelings of social rejection (Williams et al., 2000; McQuaid et al., 2015).

There are also reports on the putative role of polymorphisms in AVPR genes and mood disorder spectrum (Dempster et al., 2007). In a Swedish and a Belgian study, for instance, the haplotype defined by alleles A-T-C-A-G for the *AVPR1b* SNPs s1-s2-s3-s4-s5 was significantly over-represented in controls vs. patients with depression (Van West et al., 2004). Despite these associations, there are reports that, at least for *OXTR*, polymorphisms do not alter antidepressant treatment resistance, response or remission, nor are they associated with variations in the inflammatory pathways that have been reported to play a role in antidepressant efficacy, such as cyclooxygenase-2 and *OXTR* SNPs rs53576 and rs2254298 (Mendlewicz et al., 2012).

##### Stress

Thompson et al. (2011) found, in a sample of Caucasian girls, that subjects who were heterozygous for the *OXTR* rs2254298 polymorphism (presenting A–G substitutions), and had high early life adversity, showed the highest levels of depression and of physical and social anxiety. Another study showed influences of the rs53576 SNP on stress: Thompson and Holman (2013), for instance, found that individuals homozygous for the rs53576 G allele that had been exposed to a history of negative social environment (specifically had been suffering high economic stress, measured using a Likert scale created for the study) showed elevated post-traumatic stress symptoms after the 9/11 events in the United States, as measured using the post-traumatic stress disorder (PTSD) Checklist. The same risky G allele was associated with lower cortisol responses to stress after social support, compared to similarly challenged individuals of the same genotype, but receiving no social support (Chen et al., 2011). In addition, this G allele and the *AVPR* 320 bp RS1 SSR seem to have a gender specific interaction with cortisol plasmatic levels, where women, but not men, with high levels of poststressor OXT and the G/G genotype, felt the most positive affect after the stressor; by contrast, only men with high levels of poststressor AVP and the 320 allele of the RS1 polymorphism reported more poststressor anger than noncarriers (Moons et al., 2014).

##### Harm avoidance

In females, the A/A genotype in rs53576 is associated with an increase in harm avoidance. In addition, females with this genotype presented significantly smaller amygdalar volumes, bilaterally, especially its centromedial subregion. In addition, the A/A allele is associated with reduced resting-state functional bilateral coupling between the prefrontal cortex and amygdala: in the left hemisphere this coupling was positively correlated with harm avoidance scores in female subjects (Wang et al., 2014).

##### Neuroticism

Carriers of the G variant of rs53576 showed lower striatal DAT availability and a negative correlation between DAT availability and OXT levels (Chang et al., 2014). Furthermore, the OXT × DAT interaction was significantly correlated with the MPI neuroticism score in the A/A group (Chang et al., 2014).

#### Polygenic approach

Do *OXTR* and *AVPR* signaling pathways interact with other transmitter systems (Sauer et al., 2013)? This question has recently been approached in studies involving both a couple of well-known *OXTR* SNPs and *AVPR* SSRs, and polymorphisms in genes from pathways known to interact with these neuropeptide systems. A difficulty of this approach is that, mainly because of pleiotropy, we often do not have a strong hypothesis regarding which are relevant candidate genes and gene pathways to consider (Plomin et al., 2008). For such cases, an increasingly popular alternative is the genome-wide association studies (GWAS), which use a dense map of markers to genotype the entire genome. This requires scanning about 500,000 SNPs or, if selecting wisely based on haplotype blocks, about 350,000 SNPs (Cardon and Abecasis, 2003; Plomin et al., 2008); for these purposes, a valuable complement is to use microarrays (Plomin et al., 2008). However, given the limited accessibility to GWAS (mainly due to their cost), and the lack of microarray chips containing the *OXTR* and *AVPR* markers reviewed here [for instance, the *OXTR* SNP rs53576 is not present on many chips currently available for autism spectrum disorders (ASD); Meyer-Lindenberg et al., 2011], selecting a couple of candidate genes and studying their interactions via polygenic association studies remains a valuable approach. In any event, there are only a few reports that have used a candidate gene polygenic approach or GWAS to investigate the effects (and functionality) of polymorphisms in *OXTR* and *AVPR* on social and pathological behaviors. These few (selected) studies are discussed below.

##### Autism

Nyffeler et al. (2014) found, in a Caucasian population, that a significant part of the risk for high functioning autism is explained by the combination of four polymorphisms: HTTLPR (a polymorphic repeat inside the gene coding for the serotonin transporter), SNP rs6311 in *HTR2A* (which encodes the serotonin receptor 5-HT2A), and the rs2254298 and rs53576 SNPs in the *OXTR*. These data provide evidence supporting a polygenic inheritance of ASD, involving both the OXT and the 5-HT pathways (Nyffeler et al., 2014). Kelemenova et al. (2010), using a sample of autistic boys in Slovakia, also focused their research on several candidate gene polymorphisms associated with autism, specifically *OXT* (rs2740204), *OXTR* (rs2228485), *GABA receptor gamma 3* (rs28431127), *neuroligin* (rs5916338), and *reelin*. The authors found only one significant association: between autism and a higher number of GGC repeats in the (GGC)n STR polymorphism of the reelin gene, in addition to finding lower reelin levels in the blood and the brain of autistic patients.

##### Emotional withdrawal

Haram et al. (2015) performed an association analysis between polymorphisms at four OXT pathway genes (*OXT, OXTR, AVP*, and *CD38*) and four areas of social psychopathology (as measured by Positive and Negative Syndrome Scale), finding an association between the A allele in the rs53576 SNP and the Emotional Withdrawal traits. However, they did not find an association between any of the genes included in the analysis and a diagnosis of psychotic disorder.

##### Oppositional-defiant disorder

In a GWAS performed in a clinical sample of children and adolescents, Aebi et al. (2015) failed to find an association between oppositional-defiant disorder and the polymorphisms DRD4 exon3 VNTR (located in exon 3 of the D4 dopamine receptor gene), HTTLPR, and seven *OXTR* SNPs. They also performed a GWAS including oppositional-defiant disorder dimensions. Controlling for factors such as age, sex, and parental abilities, the authors did not find an association between any of the variables (Aebi et al., 2015).

#### What do we know about the functional consequences of these polymorphisms?

##### Amygdala volume

For *OXTR* rs2254298, participants homozygous for the G allele were found to have smaller volumes of both left and right amygdala, posterior brain stem and dorsomedial anterior cingulate cortex, as compared to carriers of the A allele (Furman et al., 2011). In this same SNP, the A allele was positively correlated with bilateral amygdala volume: the larger the number of rs2254298 A alleles an individual had, the larger their amygdala volume. Furthermore, two three-single nucleotide polymorphism haplotypes, including the rs2254298 G allele, showed significant associations with a smaller bilateral amygdala volume (Inoue et al., 2010). These associations between *OXTR* polymorphism and amygdala volume could provide a hypothesis that explains how OXTR gene variants may increase the risk of psychopathologies (Furman et al., 2011). In addition, as stated above, the A/A genotype in rs53576 is associated with significantly smaller bilateral amygdala volumes and reduced resting-state bilateral coupling between amygdala and the prefrontal cortex, specifically in women (Wang et al., 2014).

##### Amygdala activation

Sauer et al. (2013), studying the interaction between OXT plasma levels and the dopamine system, specifically the common catechol-O-methyltransferase (COMT) val158met polymorphism (a polymorphism known to influence COMT activity and, consequently, dopamine degradation at synapses), analyzed amygdala activation after the presentation of social stimuli following placebo or OXT infusions. Their results showed no gene main effect and no gene × substance interaction, but a significant gene × gene × substance interaction. Indeed, using various social stimuli paradigms, the authors found that, when given placebo, the effect of CD38 on bilateral amygdala activation was modulated by the COMT genotype; by contrast, no such COMT genotype dependence was observed following the administration of OXT (Sauer et al., 2013). These result are consistent with the report of Baumgartner et al. (2008), who found that intranasal infusions of OXT, in addition to making subjects insensitive to unreciprocated trust (as measured with a Trust game) show reduced activation of the amygdala and the dorsal striatum when faced with social stimuli (specifically social betrayal), as compared to a placebo group (see also Hurlemann et al., 2010).

Regarding *AVPR*, Meyer-Lindenberg et al. (2009) showed that two risky alleles in *AVPR1A*, the RS1 334 bp and the RS3 320 bp alleles (which have been reported to have significant transmission to autistic probands) are associated with opposing effects on amygdala activation in an emotional face-matching task, a simple perceptual task previously described to robustly engage the amygdala. In this task, subjects must decide which one of two faces is (emotionally) identical to a target face (Meyer-Lindenberg et al., 2009). The authors found that the 334 bp RS1 allele was associated with stronger bilateral responses of the amygdala, whereas the 320 bp RS3 carriers showed a smaller activation of the left amygdala. However, there was no association between these alleles and behavioral performance (Meyer-Lindenberg et al., 2009).

##### Promoter activity

Both RS1 and RS3 showed differences in relative promoter activity, as measured in the human neuroblastoma cell line SH-SY5Y, with shorter repeat alleles of RS1 and RS3 showing decreased relative promoter activity (Tansey et al., 2011).

##### Epigenetic effects

Recent findings by Reiner et al. (2015), in a sample of 43 clinically depressed and 42 healthy female subjects, suggest epigenetic effects of the *OXTR* rs53576 genotype on the patterns of methylation in exon 1 and 2 of *OXTR*. This *OXTR* SNP moderated, for instance, the association between depression and *OXTR* exon 1 methylation level. In this exon, a decreased methylation state was associated with depression. This kind of findings becomes more important now that strong evidence suggests that specific genotypes show altered levels of DNA methylation throughout the whole genome, depending on early environment (Dadds et al., 2013; Chen et al., 2015).

### Effects of intranasal administration of OXT and AVP on psychiatric symptoms

The evidence on the effects of intranasal applications of OXT and AVP on clinical populations expressing a mental disorder is relatively sparse, and echoes the mixed findings for healthy population. However, the use of intranasal OXT for the treatment of psychiatric disorders shows some promise, particularly for treating symptoms involving deficits in social functioning, such as autism, schizophrenia, borderline personality, and social anxiety disorders (Macdonald and Macdonald, 2010; MacDonald et al., 2011; Anagnostou et al., 2012; Macdonald and Feifel, 2012; Bakermans-Kranenburg and Van Ijzendoorn, 2013; Bethlehem et al., 2013; De Berardis et al., 2013; Tachibana et al., 2013; Veening and Olivier, 2013; Cardoso et al., 2014). For instance, intranasal OXT has been shown to increase eye contact in individuals with ASD, possibly by increasing the saliency of social stimuli (Auyeung et al., 2015). Similarly, intranasal OXT, in ASD patients, improves the ability to recognize the social emotions of others, as measured both at the behavioral and neural levels (Aoki et al., 2014). In addition, OXT may selectively affect the salience and hedonic assessments of socially meaningful stimuli in subjects with ASD, and thus help social attunement (Domes et al., 2013; Gordon et al., 2013).

In individuals with Borderline Personality Disorder (BPD), OXT administration has shown that the effects may differ depending on baseline conditions, such as the participant's representations and expectations and/or an OXT system that is not working properly. For instance, Bartz et al. (2011) tested whether OXT administration improves trust and cooperative behaviors in individuals with BPD vs. healthy controls, using the Assurance Game (a variation of the Prisoner's dilemma; Kreps et al., 1982; Brosnan et al., 2011). They found that participants with BPD expected their partners to be less cooperative after administration of OXT, showing the opposite effect compared to healthy controls, where OXT infusions increase trust (Kosfeld et al., 2005; Bartz et al., 2011). In addition, intranasal administration of OXT in schizophrenic populations has shown an anxiolytic (Bell et al., 2006), antidepressant (Ozsoy et al., 2009), and antipsychotic effect (Caldwell et al., 2009; Kuehn, 2011; Macdonald and Feifel, 2012; De Berardis et al., 2013).

Surprisingly, there is very limited evidence linking OXT administration to social anxiety disorder (SAD). Guastella et al. (2009) examined the effect of OXT administration as an adjunct to therapy for SAD, finding that patients treated with OXT showed significant differences in their ratings of speech performance and speech appearance compared to patients treated with placebo. Similarly, Labuschagne et al. (2010) showed, using an emotional face matching paradigm as measure, that intranasal OXT reduced amygdala reactivity to fearful faces in participants diagnosed with SAD (Labuschagne et al., 2010). In addition, the same group showed that intranasal OXT administered to SAD participants reduced cortical hyperactivity in the medial prefrontal cortex to sad faces to a level comparable to that of controls. Taken together, these studies indicate that OXT administration modulates the fear-related neural circuitry, consistent with previous research showing pro-social effects of OXT administration in healthy individuals.

Nevertheless, and as a cautionary note, a recent meta-analysis examining the effect of intranasal OXT across various clinical samples (i.e., ASD, social anxiety, depression, obsessive-compulsive disorder, schizophrenia, borderline personality disorder, and post-traumatic stress disorder) found only a small to moderate effect on psychiatric symptomatology and social competence indicators (Bakermans-Kranenburg and Van Ijzendoorn, 2013). Moreover, Macdonald and Macdonald (2010) suggested that the context-dependent and divergent effects of OXT indicate that the effects of such OXT treatments may depend on individual differences (i.e., modulation by sex, hormonal and psychiatric status, and attachment style). The available evidence reveals that chronic administration of OXT causes mixed results, even within groups afflicted by the same disorder (Tachibana et al., 2013; Dadds et al., 2014). In addition, the effects of acute vs. chronic OXT administration have not been thoroughly studied; thus, their relative merit for the treatment of psychiatric disorders is unknown. Although promising, the specific effects of OXT on psychiatric symptomatology remain unclear.

Surprisingly, there is little research on the impact of intransal AVP administration on psychiatric symptoms. Some studies have suggested a link between AVP levels and psychiatric disorders. For instance, cerebrospinal fluid AVP levels have been positively correlated with a life history of interpersonal aggression in individuals with personality disorders (Coccaro et al., 1998). Similarly, Pitman et al. (1993) investigated the effects of OXT and AVP on the emotional response of Vietnam veterans with PTSD during personal combat imagery exercises. Responses, measured by determining skin conductance, heart rate, and electromyographic responses, showed that the group that received AVP presented higher reactivity to combat imagery relative to the OXT and placebo groups, suggesting that AVP may enhance the emotional valence of events (Coccaro et al., 1998).

In healthy populations, intranasal AVP has been shown to also improve social recognition. For example, Guastella et al. (2010a) conducted a study where healthy males were asked to view faces displaying happy, neutral, or angry expressions. Using a “surprise memory test,” they showed that participants who received AVP were more likely to remember happy and angry faces compared to participants who received placebo (Guastella et al., 2010b). These results suggest that AVP also enhances human recognition of emotionally-valenced faces.

The very limited research using intranasal administration of AVP (Coccaro et al., 1998; Guastella et al., 2010b) has, nevertheless, revealed that this neuropeptide can exert important effects on social information processing. The results discussed above largely suggest that AVP may play a role in the processing of emotionally-valenced information in both healthy and clinical population.

## Discussion

A growing body of research has found associations between alleles of *OXTR* and *AVPR* and human social and pathological behavior, such as altruism (Knafo et al., 2008), generosity (Zak et al., 2007) and aggression (Luppino et al., 2014); depression (Van West et al., 2004; Apter-Levy et al., 2013; Thompson et al., 2014), empathy (Wu et al., 2012; Luo et al., 2015a,b), autism (Wu et al., 2005; Jacob et al., 2007; Kelemenova et al., 2010; Tansey et al., 2011; Nyffeler et al., 2014), stress (Thompson et al., 2011; Thompson and Holman, 2013), and schizophrenia (Golimbet et al., 2015; Levin et al., 2015). However, these results remain preliminary, and await further studies using other populations. As a cautionary note, a recent meta-analysis performed by Bakermans-Kranenburg and van Ijzendoorn (2014), found no significant association between any of five outcomes (i.e., biology, personality, social behavior, psychopathology, and autism) and the SNPs rs53576 and rs2254298 (reviewed above), concluding that these polymorphisms failed to explain a significant part of human social behavioral diversity (Bakermans-Kranenburg and van Ijzendoorn, 2014).

### Functionality

A point to keep in mind is the old maxim “correlation does not imply causation.” This means that a robust result associating, for instance, a rs53576 polymorphism with a predisposition to depression, does not imply that there exists a causal relationship between the two variables. However, such an association does have some inherent value: if we cannot find evidence showing a causal relationship (or if this relationship does not exist at all!), the data may still provide useful information, such as pointing to a genetic region where the relevant gene is located (Plomin et al., 2008). That correlation does not imply causation also raises questions about the impact of polymorphisms on gene function described in the literature. In other words, one may ask: Do the OXTR's SNPs and AVPR's SSRs play a role in causing the differences in phenotypic traits? Or, alternatively: Do these variants lack functionality or do they just result from “spurious” correlations? Yet, recent research is showing that these polymorphisms are indeed associated with different volumes of some neural structures (Inoue et al., 2010; Furman et al., 2011), differential neuronal connectivity or activation (Meyer-Lindenberg et al., 2009; Sauer et al., 2013); differential expression of the receptors (Young et al., 1999; Knafo et al., 2008), plasmatic levels of the peptides (Moons et al., 2014), or promotor activity (Tansey et al., 2011). This suggests that neuropeptide receptor polymorphisms likely do have a functional role in normal and pathological human behavior. Anyway, there are also practical benefits to identifying individuals at risk even without knowing the mechanisms responsible for the deficit (Brookfield, 2010): for instance, there are treatments and tests available that are offered on the basis of calculated risk (Plomin et al., 2008; Brookfield, 2010).

### Thinking about normal and psychopathic behavior as a continuum

It is noteworthy that both the normal and pathological behaviors covered in this review are most certainly quantitative traits, involving interactions among more than one gene, and for which the phenotypes of interest are better described as a continuum. Consequently, the field needs to emphasize polygenic association studies where, instead of a single gene underlying a trait, there are multiple (functional) gene variants involved, each making small quantitative contributions to the final phenotype (Plomin et al., 2008; Brookfield, 2010). This notion (namely, that many genes influence complex disorders or personality traits) raises the question of why such disorders are typically diagnosed as qualitative, and not as quantitative traits, with a continuum of genetic risk. This quantitative approach allows one to formulate two models: the liability threshold model, where risk is distributed normally and disorders occur in a qualitative fashion, when a threshold is exceeded (genetic continuum with discrete phenotypes), vs. a model that proposes that phenotypes change continuously from normal to abnormal, with diagnosed cases being extreme cases that differ quantitatively, but not qualitatively (Plomin et al., 2008). The latter seems to be the best descriptor for depression and alcoholism, and even for schizophrenia. It also allows finding genes that may be associated with depression in afflicted patients and with mood changes in normal patients.

### Finally: what do we still not know?

GWAS promises to be a powerful tool for constructing the complete landscape of interacting genes but, given its current cost, have not replaced research that investigates candidate genes in polygenic association studies. To date, in addition to *OXTR* and *AVPR* SNPs and SSRs, polymorphisms in *COMT, CD38, HTTLPR, HTR2A* and *DAT* are likely to be good candidates for studying the genetic bases of human social and pathological behavior. Curiously, the *OXTR* SNP rs53576 is not present on many currently available chips that are used for genome-wide association studies of ASD (Meyer-Lindenberg et al., 2011), limiting their use for such studies.

In addition, an open challenge is to determine the exact mechanism by which neuropeptides influence psychiatric symptoms. Although some reports are shedding light on the impact of different gene polymorphisms on socially relevant behaviors and their associated psychiatric disorders, the complete pathways between genes, gene interactions and behavior is still a black dark forest. As told by Chomsky, “there's a famous joke about a drunk under a lamppost looking for a pencil dropped on the ground. Somebody comes up and asks ‘What are you looking for?’ He says, ‘I’m looking for a pencil that I dropped.' ‘Well, where did you drop it?’ He says, ‘Oh, I dropped it across the street.’ ‘Well, why are looking here?’ ‘This is where there is light.’ That's the way the sciences work. […] If you try to move it a little further, maybe ultimately you'll get across the street.”

## Author contributions

MA, MM, MR, AR, and JE had fun conceiving, drafting and critically revising this review.

### Conflict of interest statement

The authors declare that the research was conducted in the absence of any commercial or financial relationships that could be construed as a potential conflict of interest.
